# A physician assistant entry-level doctoral degree: more harm than good?

**DOI:** 10.1186/s12909-021-02725-5

**Published:** 2021-05-14

**Authors:** Violet Kulo, Shani Fleming, Karen L. Gordes, Hyun-Jin Jun, James F. Cawley, Gerald Kayingo

**Affiliations:** Physician Assistant Leadership and Learning Academy (PALLA), University of Maryland Baltimore Graduate School, 620 West Lexington Street, Baltimore, MD USA

**Keywords:** Physician assistant, Entry-level doctoral degree, Benefits and risks, Post-graduate education, Terminal degree

## Abstract

**Background:**

As most health professions in the United States have adopted clinical or practice doctorates, there has been an ongoing debate on whether physician assistants (PAs) should transition from a master’s to a doctorate as the terminal degree. The authors examined perceived risks, benefits and impact of transitioning to an entry-level PA doctoral degree.

**Methods:**

A multi-prong, mixed-methods approach was used that included a literature review and collecting quantitative and qualitative data using a survey and interviews. Bivariate analysis and binomial logistic regression were performed to evaluate relationships between perceptions/perspectives on an entry-level PA doctoral degree and the anticipated impact of it causing more harm than good to the PA profession. Deductive content analysis was used to analyze the qualitative data.

**Results:**

Of 636 PA clinicians and students (46% response rate), 457 (72%) disagreed that an entry-level PA doctoral degree should be required. More than half of the respondents (54%) agreed that it should be offered but not required and 380 respondents (60%) agreed that an entry-level doctoral degree would cause more harm than good. Race, educational attainment, occupation, and length of practice as a PA were significantly associated with having a perception of causing more harm. There was strong positive association between the perception of a doctoral degree causing more harm with expectations of having a negative impact on the availability of clinical training sites (OR = 4.39, *p* < .05). The most commonly cited benefits were parity with other professions and competitive advantage, whereas the perceived risks were increased cost for education, decreased diversity in the profession, and negative impact on the PA/physician relationship.

**Conclusions:**

The major takeaway of our study was that perceived benefits and risks are strongly influenced by the lens of the stakeholder. While the majority of PAs and students appear to be not in favor mainly due to the potential harm, the proportion of those in favor is not insignificant and their views should not be ignored. Addressing concerns with key stakeholders could help the PA profession to transition to a doctoral degree with minimal adverse impact.

## Background

For over two centuries, the doctor of medicine (MD) and the doctor of osteopathic medicine (DO) have been the terminal degrees in the field of medicine. More recently, other health professions in the United States (US) such as physical therapy, pharmacy, and advanced practice nursing have adopted entry-level clinical doctorates [1–3]. To date, physician assistants (PA) are the only prescribing practitioners who have not transitioned to an entry-level doctorate [4]. While the PA profession is an emerging profession internationally [5], this is an established field in the US that provides substantial contribution to the healthcare workforce in all medical specialty areas. PAs are medical professionals who diagnose illness, develop and manage treatment plans [6]. Within this profession, a master’s degree is considered the terminal degree; however, there has been an ongoing debate whether PAs should transition to an entry-level doctorate.

The debate on PA doctoral education was initially addressed at the 2009 PA Clinical Doctorate Summit conducted by the Physician Assistant Education Association (PAEA) and the American Academy of PAs (AAPA). The purpose of the summit was to address the question of whether a clinical doctorate was the appropriate entry-level degree for the profession and subsequently endorsed the master’s degree as the entry-level and terminal degree for the profession [7–9]. Post-professional doctorates such as the doctor of medical science (DMSc) were supported as an educational option for PAs as long as they were not PA-specific [10]. While the PA profession has remained consistent in not endorsing a PA entry-level doctorate, there has been renewed interest in the PA doctoral education debate and recently PAEA commissioned research to investigate the feasibility of an entry-level doctoral degree for PAs [11].

The desire to transition to an entry-level doctoral degree by various health professions was motivated by specific practice goals. Expanding scope of practice and advancing clinical competency were key motivators for physical therapy and nursing [1, 2, 12]. Questions remain if these motivating factors are right or even exist for the PA profession. PAs have traditionally practiced team-based care under the supervision of a physician. A concern for some PAs is that a PA entry-level doctoral degree could signal abandoning team-based practice in favor of greater autonomy and disrupt the traditional symbiotic relationship between PAs and physicians [9]. In addition, since the PA scope of practice is not typically a function of formal educational level, many question if additional doctoral level training would enhance the clinical competency of PAs. As noted in other professions who have transitioned to an entry-level doctoral degree, there are also concerns about increased debt burden, return on investment, reduced net present value relative to physicians, workforce supply, and diversity [13–15]. Literature on the impact of an entry-level doctoral degree in nursing, pharmacy, and physical therapy revealed an increase in program length and cost, improved opportunites for leadership, but no significant change in scope of practice or in patient-related outcomes [16–18].

Rapid shifts in the health care environment, changing demands in clinical care, and the explosion of available knowledge require transformation in the way healthcare education is delivered to assure quality patient outcomes [19–21]. Transitioning to doctoral level education has been one method by which different professions have responded to these challenges. Within the PA profession, emerging discussions on PA Optimal Team Practice (OTP), the increase in number of professions conferring clinical doctorates, and the increasing number of PAs seeking post-professional doctorates have reinvigorated the PA doctoral degree debate [22–24]. Some have expressed a need for PAs to transition to a doctoral degree to achieve parity with other health professions such as the nurse practitioners and that transitioning to a doctoral degree could increase career opportunities for PAs, especially in areas of leadership, academia and policy [24]. For the PA profession to move forward in addressing the doctoral degree debate, data showing stakeholders’ perceptions are important to assess the perceived risks, benefits and impact both internal and external to the PA profession. In this paper, we provide the results of a mixed-methods study assessing views across various interprofessional stakeholders on the perceived risks, benefits and impact of transitioning to an entry-level PA doctoral degree.

## Methods

### Study design

This investigation used a multi-prong, mixed-methods approach, involving cross-sectional survey data and semi-structured interviews to capture stakeholders’ views on and impact of transitioning to an entry-level PA doctorate. Survey study participants were recruited through two mechanisms, AAPA’s PA Observations Service [25] and the Maryland Academy of Physician Assistants listserv. The survey was distributed to a national sample of 1368 PA clinicians and students and a total of 636 surveys were completed. Results of power analysis using G*Power [26] software package indicated the estimated sample size to be at least 74 and 160 subjects to obtain 95% chance of detecting large- (*d* = .35) and medium-sized effect (*d* = .15) [27], respectively as significant at the .05 level. Surveys were distributed in June 2020 through an email with a link to Qualtrics (Provo, Utah), a private survey research company specializing in online surveys. The survey was available for 1 week, and participants received two email reminders to complete the survey.

For the semi-structured interviews, we recruited a purposive sample of interprofessional stakeholders including clinical partners (i.e., practicing clinicians – PAs and physicians); non-PA academic leaders (i.e., Deans, Provosts, Presidents - across the health professions of nursing, medicine, pharmacy, and physical therapy); PA education community members (i.e., PA association leaders and members, PA Program Directors, PA faculty), and PA employers. With a total of 38 participants, the semi-structured interviews ranging from 30 to 60 min were conducted via Zoom or telephone in June and July of 2020. The University of Maryland, Baltimore Institutional Review Board approved this study. Informed consent was obtained from all participants.

### Measures/instruments

The development of the survey instrument and interview guide were grounded in the results of an interprofessional literature review examining the impact of the doctoral transitions for nursing, pharmacy and physical therapy professions. Key concepts within the overarching themes of perceived risks prior to their transition and impact after their transition were identified from this literature review and used to generate the questionnaires for both the quantitative survey and qualitative interviews. The questions were arranged by overarching themes including, benefits and risks, impact on the profession, impact on patient-related outcomes, and impact on scope of practice. The survey instrument was beta tested by administering it to a pilot group of PA faculty, students, and clinicians prior to distributing it nationally to ensure the instrument’s face validity and reliability. Recommendations were used to refine the survey prior to distribution to study participants.

The survey consisted of 28 items, using a Likert Scale and open-ended questions. The survey collected responses on demographic information (i.e., sex, ethnicity, race, educational attainment, occupation, length of practice as PA) and respondents’ perspectives on the risks and impact of transitioning to an entry-level PA doctoral degree.

The interview guide was comprised of 11 questions, of which eight were common across all stakeholders and three were specific to each stakeholder group. The interview questions asked interviewees to provide commentary on the risks of an entry-level doctoral degree, as well as potential impact on the profession.

### Data analysis

#### Quantitative

To analyze the survey data, the six survey response categories were coded into three categories as follows: for questions on the Likert scale of disagree to agree, the coding was (0) for disagree [combining strongly/somewhat disagree], (1) for neutral, and (2) for agree [combining strongly/somewhat agree]. For questions related to feasibility, the coding was (0) for not feasible and (1) for feasible [combining slightly/moderate/very/extremely feasible].

Analyses were conducted using SPSS version 26 for Windows (IBM Corp, Armonk, New York). Missing data (ranging from 0.2 to 0.9%) were handled using listwise deletion due to the low frequency of missing values. We performed descriptive statistics to define the characteristics of the sample and evaluate the main study variables. Bivariate analyses using chi-square tests assessed the relationships between independent variables and the dependent variable. The independent variables were demographics, perception on requiring/offering a PA doctoral degree, feasibility to transition, perspectives on negative impact to the PA-physician relationship and the availability of clinical training sites. The dependent variable was the perception of the entry-level doctoral degree causing more harm to the PA profession. Binomial logistic regression analyses were used to test for associations between perception of transitioning to an entry-level doctoral degree and causing more harm to the PA profession using two models. Model 1 tested the association between demographic covariates and the perception of the entry-level doctoral degree. The main study variables (i.e., to be required, to be offered, feasibility, negative impact) were added in Model 2 to investigate their association with the perception of the entry-level doctoral degree causing more harm after adjusting for demographic covariates.

#### Qualitative

We used a phenomenological approach to explore the lived experiences of participants [28]. The interview data and free-text responses from the survey were analyzed using an iterative process and deductive content analysis with pre-determined codes [29]. Three researchers independently analyzed the free-text responses with reference to pre-determined (a priori) themes derived from the comprehensive interprofessional literature review: risks and impact on the PA profession. Coding output from each reviewer was compared. Differences were discussed until a consensus was reached to ensure inter-rater reliability.

## Results

Out of a sample of 1368 practicing PAs and PA students, 636 consented and responded to the survey (46% response rate). Forty eight states were accounted for in the national responses received, as well as District of Columbia and Puerto Rico. The demographic characteristics of the sample size are representative of the national PA population. Table 1 presents the demographic characteristics based on the number of participants who responded to each question.

**Table 1:** Respondents’ demographic characteristics

	*n*^a^	*%*
Sex		
Female	433	68.5
Male	199	31.5
Hispanic/Latinx		
Yes	47	7.4
No	584	92.6
Race		
White	523	82.2
Black/African American	39	6.1
American Indian/Alaska Native	6	0.9
Asian	41	6.4
Native Hawaiian/Pacific Islander	2	0.3
Multirace	18	2.8
Others	19	3.0
Educational attainment		
Associate degree	2	0.3
Bachelor’s degree	97	15.3
Master’s degree	475	74.9
Doctorate degree	60	9.5
Occupation^b^		
Student	109	17.2
PA clinician	524	82.8
Length of practice as PA		
Current student	107	16.9
< 5 years	172	27.1
5–10 years	148	23.3
11–25 years	169	26.7
> 25 years	37	5.8
Non-PA	1	0.2

Note. ^a^Variations existed in the number of respondents by question. ^b^Some of the PA clinicians reported additional professional roles such as faculty and administrators but they were all counted as PA clinicians

A majority of respondents were female (*n* = 433, 69%), non-Hispanic/non-Latinx (*n* = 584, 93%), and White (*n* = 523, 82%). Most respondents held a master’s degree (*n* = 475, 75%), followed by bachelor’s degree (*n* = 97, 15%), doctoral degree (*n* = 60, 10%), and associate degree (*n* = 2, 0.3%). A total of 109 (17%) respondents identified themselves as PA students, and the remainder as practicing PAs (*n* = 524, 83%). Length of practice ranged from less than 5 years (*n* = 172, 27%) to more than 25 years (*n* = 37, 6%). The 38 participants who took part in the semi-structured interviews comprised 19 PA association leaders and members (50%), 9 PA program directors and faculty (24%), 6 non-PA academic leaders (16%), 2 physicians (5%), and 2 employers (5%). A majority of interviewees (*n* = 25, 66%) were male.

### Quantitative results

**Fig. 1:**
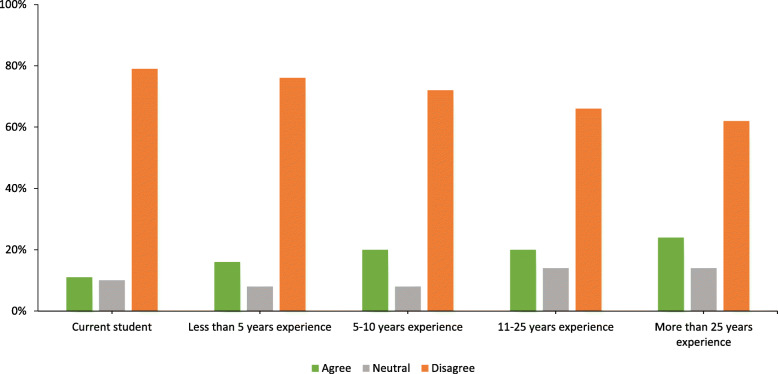
Percentages of respondents by years as a PA on question of requiring an entry-level doctoral degree

**Fig. 2:**
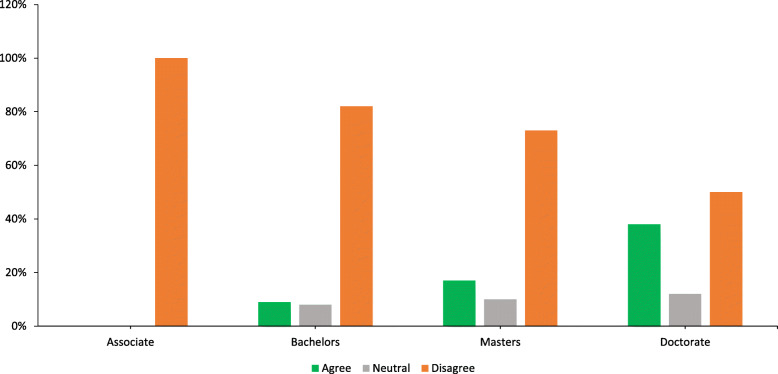
Percentages of respondents by highest degree attained on question of requiring an entry-level doctoral degree

When respondents were asked if the entry-level doctoral credential should be required for PAs, both the majority of practicing PAs and students (*n* = 457, 72%) disagreed but the extent of disagreement varied between PA clinicians (*n* = 369, 79%) and PA students (*n* = 86, 88%). For clinicians, length of professional experience and highest earned academic degree impacted their response to this question, with fewer years of experience (see Fig. 1) and lower degree (see Fig. 2) tending towards opposition to requiring a doctoral transition.

Notably, when asked if the entry-level doctoral degree should be offered, but not required the majority (*n* = 341, 54%) of respondents (PAs and PA students) agreed with this statement, 73 (12%) respondents were indifferent, and a total of 221 (35%) combined PAs and PA students disagreed. For those supporting offering but not requiring an entry-level doctoral degree, a majority were practicing clinicians (*n* = 289, 55%) versus students (*n* = 41, 45%) who, remained against a doctoral transition.

The overwhelming disagreement with the profession moving toward an entry-level doctorate was consistent with the final question of the survey asking if the entry-level doctorate would do more harm than good to the profession. Notably, more than half of the respondents agreed that transitioning to an entry-level doctoral degree may cause more harm than good to the PA profession (*n* = 380, 60%) see Table 2.

**Table 2:** Descriptive statistics on perception of the pa entry-level doctoral degree

	*n*	*%*
The PA doctoral degree to be required		
Disagree	457	72.0
Neutral	65	10.2
Agree	113	17.8
The PA doctoral degree to be offered but not be required		
Disagree	221	34.8
Neutral	73	11.5
Agree	341	53.7
Feasibility to transition to an entry-level doctoral degree		
Not feasible	128	20.2
Feasible	507	79.8
Negative impact to the PA-physician relationship		
Disagree	180	28.3
Neutral	139	21.9
Agree	317	49.8
Negative impact to the availability of clinical training sites (rotations)		
Disagree	146	23.2
Neutral	150	23.8
Agree	334	53.0
More harm of the entry-level of doctoral degree to the PA profession		
Disagree	159	25.1
Neutral	94	14.8
Agree	380	60.0

Approximately one-fifth of the sample responded that transition from the PA profession to an entry-level doctoral degree would not be feasible at all (*n* = 128, 20%). About 5 in 10 of the respondents agreed that an entry-level PA doctoral degree will negatively impact the PA-physician relationship (*n* = 317, 50%) and the availability of clinical training sites (*n* = 334, 53%).

Table 3 presents bivariate relationships regarding perception of an entry-level doctoral degree causing more harm to the PA profession by demographics and the main study variables.

**Table 3:** Bivariate analyses regarding perception of the entry-level doctoral degree causing more harm to the PA profession

	Disagree		Neutral		Agree		*χ*^*2*^ *(df)*	*p*
	*n*	*%*	*n*	*%*	*n*	*%*		
Sex								
Female	97	22.4	66	15.2	270	62.4	4.56 (2)	.10
Male	60	30.3	28	14.1	110	55.6		
Hispanic/Latinx								
Yes	15	31.9	5	10.6	27	57.4	1.60 (2)	.45
No	144	24.6	89	15.2	353	60.2		
Race								
Black/African American	10	25.6	13	33.3	16	41.0	12.07 (2)	.00
Non-Black/non-African American	149	25.1	81	13.6	364	61.3		
Educational attainment								
Master’s degree or lower	130	22.6	88	15.3	356	62.0	19.98 (2)	.00
Doctorate degree	29	49.2	6	10.2	24	40.7		
Occupation^a^								
Student	23	21.1	8	7.3	78	71.6	8.60 (2)	.01
PA clinician	136	26.0	85	16.3	302	57.7		
Length of practice as PA^b^								
< 5 years	30	17.4	32	18.6	110	64.0	9.57 (2)	.01
≥ 5 years	106	30.0	54	15.3	193	54.7		
To be required								
Disagree	47	10.3	57	12.5	351	77.1	242.54 (2)	.00
Agree	87	77.7	17	15.2	8	7.1		
To be offered but not be required								
Disagree	32	14.5	10	4.5	179	81.0	86.87 (2)	.00
Agree	121	35.7	77	22.7	141	22.7		
Feasibility of transition								
Not feasible	0	0.0	1	0.8	127	99.2	102.35 (2)	.00
Feasible	159	31.5	92	18.3	253	50.2		
Negative impact to the PA-physician relationship								
Disagree	108	60.3	26	14.5	45	25.1	198.72 (2)	.00
Agree	19	6.0	29	9.2	268	84.8		
Negative impact to the availability of clinical training sites								
Disagree	90	62.1	21	14.5	34	23.4	167.43 (2)	.00
Agree	31	9.3	30	9.0	272	81.7		

Note. ^a^Other than students or PA clinicians (faculty, hospital administrator, higher education administrator, PA leader, and non-PA clinician) were excluded based on the distribution

^b^Current students and non-PAs were excluded for data analytical purpose

Among demographics, race [*χ*^*2*^ (1, *N* = 633) = 12.07, *p* < .001], educational attainment [*χ*^*2*^ (1, *N* = 633) = 19.98, *p* < .001), occupation [*χ*^*2*^ (1, *N* = 632) = 8.60, *p* < .05], and length of practice as PA [*χ*^*2*^ (1, *N* = 525) = 9.57, *p* < .05] were significantly associated with perception of an entry-level doctoral degree causing more harm than good to the PA profession.

Specifically, Non-Black/Non-African American respondents, those who held a master’s degree or lower, students, and PAs who have practiced for less than 5 years were more likely than their counterparts to agree that the entry-level PA doctoral degree would cause more harm to the PA profession. Additionally, respondents who disagreed with requiring [*χ*^*2*^ (1, *N* = 632) = 242.54, *p* < .001] or offering [*χ*^*2*^ (1, *N* = 632) = 86.87, *p* < .001] an entry-level doctorate were more likely to perceive more harm to the PA profession compared to those who agreed with requiring or offering the degree.

Those who responded that it is not feasible for the PA profession to transition to an entry-level doctoral degree were more likely than those who reported that it is feasible to perceive more harm of entry-level PA doctoral degree to the PA profession [*χ*^*2*^ (1, *N* = 632) = 102.35, *p* < .001]. Respondents who expected negative impact to the PA-physician relationship [*χ*^*2*^ (1, *N* = 633) = 198.72, *p* < .001] and the availability of clinical training sites [*χ*^*2*^ (1, *N* = 628) = 167.43, *p* < .001] were more likely to agree with causing more harm to the PA profession compared to respondents who did not expect a negative impact.

Table 4 presents the results of the binomial logistic regression analysis displaying odds ratios for those who agreed that an entry-level doctoral degree would cause more harm to the PA profession, with those who disagreed as a reference group.

**Table 4:** Binomial logistic regression model regarding perception of the entry-level doctoral degree causing more harm to the PA profession

Model	OR	95% CI	*p*
Model 1			
Black/African American^a^	.45	.11–1.78	.26
Doctorate degree^b^	.35	.16–.78	.01
≥ 5 years^c^	.72	.37–1.39	.32
Model 2			
Black/African American^a^	.28	.03–2.32	.24
Doctorate degree^b^	.82	.22–3.13	.77
≥ 5 years^c^	.54	.19–1.55	.25
To be required^d^	.07	.02–.21	.00
To be offered but not be required^d^	.38	.13–1.11	.08
Feasibility to transition^e^	.00	.00	1.00
Negative impact to the PA-physician relationship^d^	2.43	.80–7.38	.12
Negative impact to the availability of clinical training sites ^d^	4.39	1.57–12.32	.01

Model evaluation: Model 1 *R*^*2*^ = .06; Model 2 *R*^*2*^ = .72.

Note. *OR* odds ratio, *CI* Confidence interval

Reference group for predictors: ^a^Non-black/non-African American, ^b^Master’s degree or lower, ^c^ ≤ 5 years, ^d^disagree, ^e^not feasible. Since there was no variance in the category variable of occupation, that variable was removed from the regression analysis. Due to a small sample size, neutral category was excluded for the data analytic purpose

Regression Model 1 assessed how demographic characteristics are associated with the perception that an entry-level doctoral degree would cause more harm. Respondents who held a doctorate degree (OR = .35, 95% CI = .16–.78, *p* < .05) tend to disagree with an entry-level PA doctoral degree causing more harm to the PA profession. Model 2 included other variables related to the perception/perspectives of an entry-level PA doctoral degree (i.e., requiring, offering, feasibility, negative impact to the PA-physician relationship and the availability of clinical training sites.)

Respondents who agreed that the entry-level PA doctoral degree should be required (OR = .07, 95% CI = .02–.21, *p* < .001) tend to disagree with it causing more harm to the profession. Expectations of having a negative impact on the availability of clinical training sites (OR = 4.39, 95% CI = 1.57–12.32, *p* < .05) has strong positive association with the perception of an entry-level doctoral degree causing more harm while adjusting for covariates.

**Fig. 3:**
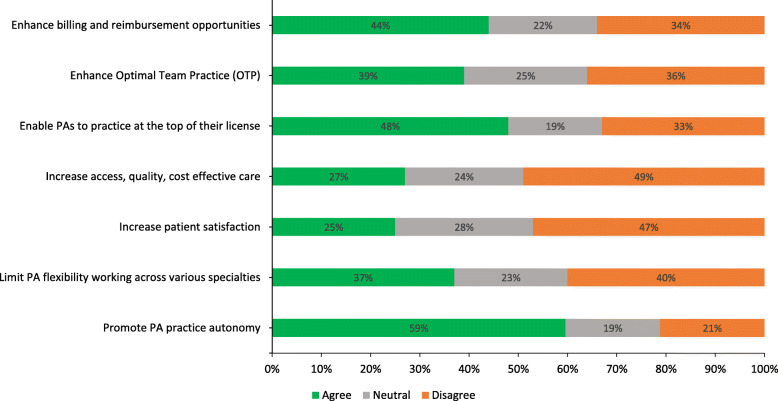
Percentages of respondents on question of how an entry-level doctoral degree might impact PA scope of practice and patient-related outcomes

When respondents were asked about the potential impact of transitioning to a doctoral degree on the PA scope of practice and patient-related outcomes, responses varied depending on the aspect of practice and outcome in question (see Fig. 3).

Positive impacts included, enhanced billing and reimbursement opportunities (*n* = 277, 44%), enhanced optimal team practice (*n* = 248, 39%), enhanced capacity for PAs to practice at the top of their license (*n* = 305, 48%), and the possibility of enhancing practice autonomy (*n* = 374, 60%). The majority of respondents agreed that a doctoral transition would not: increase access, quality and cost effectiveness of care (*n* = 308, 49%) or increase patient satisfaction (*n* = 297, 47%). Regarding impact on flexibility, respondents (*n* = 249, 40%) indicated agreement a transition would not impact PA flexibility for working across specialties.

**Fig. 4:**
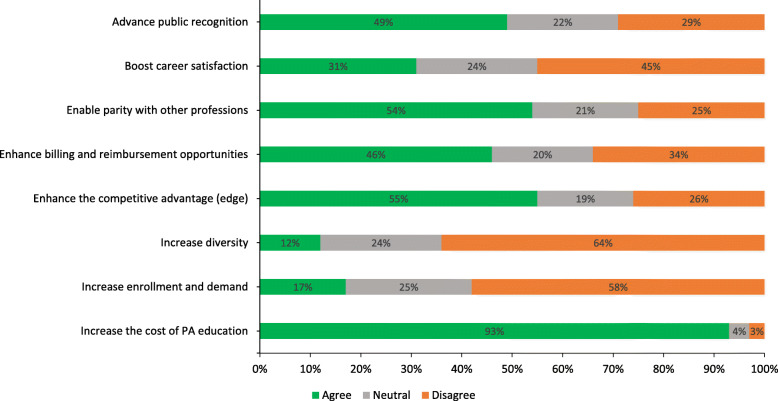
Percentages of respondents on question of how an entry-level doctoral degree might impact the PA Profession

When respondents were asked about the potential impact of transitioning to a doctoral degree on the impact of the PA profession as a whole, responses also varied depending on the category item (see Fig. 4).

Positive impacts included, advance public recognition (*n* = 309, 49%), parity with other professions (*n* = 342, 54%), enhanced billing and reimbursement opportunities (*n* = 289, 46%), and enhanced competitive advantage (*n* = 345, 55%). Negative impacts included, increase to the cost of PA education (*n* = 585, 93%) and negative impact on diversity (*n* = 403, 64%). Many respondents agreed that a doctoral transition would not increase enrollment and demand (*n* = 367, 58%) or boost career satisfaction (*n* = 284, 45%).

When respondents were asked to rank aspects of the PA profession that would either be positively or negatively impacted by a doctoral transition, the top three positive impacts were leadership opportunities; parity with other professions; and competitive advantage (edge), whereas the top three ranked negative impacts were cost of PA education programs; diversity in the PA profession; and PA-physician relationship.

If the PA profession opted to have a doctoral degree as the terminal agree, 73 respondents (12%) supported a Bachelor’s to Doctorate pathway, 113 (18%) supported a Master’s to Doctorate pathway and 435 (70%) supported a bridge program (working clinically) to doctorate. When exploring title nomenclature for a PA doctoral degree, respondents reported that the most appropriate title from the choices provided was Doctor of Medical Science (DMSc, 24%), followed by Doctor of Physician Assistant Studies (DPAS, 22%), Doctor of Medical Science (DMS, 21%), Doctor of Physician Assistant Practice (DPAP, 11%), Doctor of Physician Assistant (DPA, 11%), Doctor of Science in Physician Assistant Studies (DScPAS, 7%), with the least appropriate title being the Doctor of Health Science (DHSc, 4%).

### Qualitative results

Using a deductive approach, we analyzed the semi-structured interview data and the responses to the open-ended survey questions. The following perceived risks and impact of a doctoral transition were expressed by various stakeholders, see Table 5 for sample responses.

**Table 5:** Perceived risks and impact expressed by stakeholder

	Perceived Risks	Perceived Impact on PA Profession
Practicing PAs	“Requiring an entry-level PA doctoral degree will do more harm than good by increasing the cost of PA education, subsequently increasing the cost of patient care. The increase in student loan debt will drive more PAs to work in high-paying specialties, decreasing the number of PAs in primary care, further increasing the shortage of primary care providers in our health system.” “If adopted, this decision would be detrimental to the trust and understanding that has just recently been adopted in the patient community. It will cause a significant barrier in healthcare delivery, both financially and geographically. It will also change the landscape of admission to the profession, forsaking the heart of the individuals who love the mission and vision of, and who are called to, the physician assistant profession.”	“I think that the entry level PA doctoral degree is needed to keep parity with the NP profession. I have experienced firsthand the disadvantage when applying for jobs against NPs. I think the doctoral degree will provide needed leverage to push of autonomous practice which is long overdue.” “I see a doctoral degree as beneficial for the non-clinical aspects of being a PA. Informatics, MSL, research, leadership. It should not impact clinical practice unless there is a way to practice without a SP if one has a doctorate.”
PA Students	“…. What would be the difference between PAs and physicians at that point? The public would be confused, and it could damage the physician relationship if they believe we are trying to be like them or replace them. The doctoral degree could do some good but overall I think it will hurt the profession in too many ways for it to be worth it.” “Increasing length and cost of training will likely diminish diversity of the PA profession and result in further deviation from primary care.”	“I feel this this the only feasible step toward ensuring optimal team practice and parity with DNPs.” I do see potential with those who decide to pursue a doctorate after being in clinical practice - it allows a pathway for continued professional growth and future opportunities as a PA (our career ladder would not stop at the clinical level or be as limited), however this should not be the required entry level degree for our profession.
Clinical Partners (i.e., Physician Practitioners)	“…. This is more about independent practice like NPs want to do in some states, but it needs to be interdependent. Interdependence is of higher value than independence. The only way the whole planet will survive is if we are more interdependent.” “.... no return on investment, increase in clinical role/salary unlikely post transition.”	“I don’t anticipate change in scope of practice.” “There are no benefits in terms of the PA profession now. The master’s degree is very appropriate.”
Academic Leaders (i.e., Dean, Provosts, Presidents - Across the health professions)	“PAs with a master’s degree may feel marginalized.” “Be prepared for push-back from other professional groups like medicine and a subset of population within PA profession”	“No anticipation for change in scope of practice, there is potential for increased role within academic settings and research capacity.” “It will put the PA profession on par with other allied health professionals that have transitioned.”
PA Association Leaders and Members	“It is a high risk to the profession. PAs may no longer be a value add for patient care.” “The master’s degree is appropriate, otherwise PA profession will be less accessible and out of reach for those we need to enter the profession.”	“If the efforts to diversify the profession are distracted by a doctoral degree then it should not be endorsed.” “A doctoral degree will enhance the image of PAs and level the playing field with the other health professions, there will be professional parity.”
PA Program Directors and Faculty	“Faculty shortage will likely increase.” “….. A doctoral degree is taking us from away from original mission.” “This will create a barrier to workforce supply.”	“If there are more hoops to jump through it might drive applicant pool down, but I am not sure if it will affect the diversity of the applicants.” “It will have more leadership opportunities, such as clinical managers or clinical/medical directors.”
PA Employers	“They cannot refer to themselves as doctor, there is risk of misrepresentation.” “…. Will it decrease flexibility of the profession?” “This will confuse patients.”	“This may challenge the cost savings of PAs as the pay difference between MDs and PAs is decreasing.” “PAs with advanced degree may benefit from increase in patient preference.”

#### Perceived risks

Participants expressed concern that a doctoral degree transition might harm the PA-physician relationship, confuse patients, and defeat the purpose of having advanced practice providers that are mid-level general practitioners. There was also consideration that it would potentially marginalize PAs with master’s degrees. More concerns included the potential increase in cost of education and student debt, possibility of a longer curriculum, and an increase in faculty shortage. These concerns were seen as a potential threat to building diversity within the profession as well as reducing overall PA career flexibility. Stakeholders commented on how the PA profession has served as a cost savings for the health care system and contemplated how a doctoral degree transition, resulting in potential higher salaries for entry-level graduates, would dissipate this cost savings. Further, concern included that an entry-level doctoral requirement would be misaligned with the historical roots of the PA profession. Noting, any increases in the length of entry-level education would defeat the original mission and design of PA education to provide a quick supply of healthcare workforce.

#### Perceived impact on PA profession

Responses directed at how this transition would impact the PA profession, included comments that a doctoral degree would put PAs on a level playing field with NPs and enable PAs to remain competitive in healthcare. Several stakeholders stated a belief that the current master’s degree is the most appropriate terminal degree and were concerned about how a transition would affect PAs already practicing. Some participants felt a doctoral degree would have no immediate return on investment with no change in patient perception, clinical expertise or impact on the scope of PA practice. However, other participants felt that a doctoral degree would open the scope of PA practice in states where PAs are more limited. A subset of those interviewed, primarily individuals identifying as having a primary role in higher education, suggested a doctoral transition could increase leadership opportunities within education and research. Participants also noted that having a doctoral degree would allow PAs access to administrative positions in the healthcare system and entry into policymaking and regulation.

## Discussion

As nearly all health professions in the US have adopted or are considering clinical or practice doctorates, there has been an ongoing debate on whether PAs should transition from a master’s to a doctorate as the terminal degree for the profession. This study investigated the perceptions of various stakeholders regarding risk and impact of transitioning to an entry-level PA doctoral degree. Based on a comprehensive analysis of all of the data obtained, our main finding is that the perception surrounding risks/benefits is strongly influenced by the lens of the stakeholder. While University and PA leaders, as well as other healthcare professionals who have already transitioned to a clinical doctorate believe an entry-level PA doctoral degree should be supported in some format, the majority of practicing PAs and students disagree with a transition to an entry-level doctoral degree. Our findings are consistent with previous studies completed about 10 years ago, which found that PAs were not supportive of an entry-level doctorate for the PA profession [13, 30, 31]. For our survey population, we found that the majority of PAs are concerned that transitioning to an entry-level doctoral degree would do more harm than good to the PA profession. Over four-fifths of the survey population disagree that an entry-level doctoral degree should be required. However, a significant number of respondents were supportive if the doctoral degree was offered as an option but not required, while some disagree to even the option of an offering of the degree.

Despite the perception that doctoral degrees are prestigious and will attract scholastically advanced students, many have voiced concerns regarding potential risks in a transition. Those not in favor of a transition believe it would increase the length and cost of PA education, decrease diversity in the profession, impose a negative impact on the PA/physician relationship, decrease the profession’s flexibility, and cause confusion for patients. There is also concern regarding exacerbating faculty and clinical site shortages, and adversely impacting the supply of PA workforce. The opportunity cost of an entry-level doctoral degree for PAs as well as the point at which such additional education would reach diminishing marginal returns remains to be established.

For those who support an entry-level PA doctorate, the anticipated benefits are in areas of professional development, leadership, research, clinical competency scope of practice and autonomy [9]. Results from this study suggest that an entry-level PA doctoral credential might improve parity and competitive advantage, but there were no significant perceived benefits in improving scope of practice or patient outcomes. Further comprehensive studies are warranted to ascertain generalizable perspectives from various stakeholders. Outside the PA profession, several medical schools are switching from the traditional 4 years to accelerated 3 year MD and DO programs [32–34]. As the healthcare system continues to evolve rapidly, there is a critical need to examine the credentials and competencies of the health workforce [20, 35]. To our knowledge, this is one of the few studies that addresses this issue of doctoral education credential in the PA profession. The strengths of our study include using a large national sample size and employing mixed-methods approach to ensure data triangulation. The study was, however, limited by the fact that some key stakeholder groups such as physicians and some demographic groups such as African Americans were under-represented in both the survey and interviews. There were also variations in the number of respondents by question for the quantitative survey. Causal relationships among perceptions/perspectives of the entry-level doctorate cannot be established due to cross-sectional design of this study.

## Conclusions

Taken together, there are both significant benefits and risks for the PA profession in transitioning to a doctorate as the terminal credential. While some respondents anticipate that there will be benefits, the overall majority of respondents believe it will do more harm than good. As the profession explores the next best step, it is imperative to do so thoughtfully and proactively. This study suggests the PA profession should continue to cultivate relationships and address concerns with key stakeholders. Knowledge of perceived risks and potential threats positions the profession to transition to a PA doctoral degree with minimum adverse impact. A systematic approach to PA doctoral education should not just follow other health professions, it should be tailored to the unique features of the PA profession and their roles in team-based practice and the changing healthcare milieu.

## Data Availability

The datasets used and/or analyzed during the current study are available from the corresponding author on reasonable request.

## Abbreviations

AAPA: American Academy of Physician Assistants
DO: Doctor of Osteopathic Medicine
MD: Doctor of Medicine
OTP: Optimal Team Practice
PA: Physician Assistant
PAEA: Physician Assistant Education Association
